# Missed Appointments at a Child Development Centre and Barriers to Access Special Needs Services for Children in Klang Valley, Malaysia: A Mixed Methods Study

**DOI:** 10.3390/ijerph19010325

**Published:** 2021-12-29

**Authors:** Fariza Fadzil, Idayu Badilla Idris, Norazlin Kamal Nor, Juriza Ismail, Azmi Mohd Tamil, Kamaliah Mohamad Noh, Noraziani Khamis, Noor Ani Ahmad, Salimah Othman, Rohana Ismail

**Affiliations:** 1 Department of Community Health, Faculty of Medicine, Universiti Kebangsaan Malaysia, Jalan Yaacob Latif, Bandar Tun Razak, Cheras, Kuala Lumpur 56000, Malaysia; farizafadzil@yahoo.com (F.F.); drtamil@ukm.edu.my (A.M.T.); 2 Family Health Development Division, Public Health Department, Ministry of Health Malaysia, Complex E, Precinct 1, Putrajaya 62590, Malaysia; salimah_othman@moh.gov.my (S.O.); rohana.ismail@moh.gov.my (R.I.); 3 Department of Paediatrics, Faculty of Medicine, Universiti Kebangsaan Malaysia, Jalan Yaacob Latif, Bandar Tun Razak, Cheras, Kuala Lumpur 56000, Malaysia; norazlyn@ppukm.ukm.edu.my (N.K.N.); juriza@ppukm.ukm.edu.my (J.I.); 4 Faculty of Medicine, University of Cyberjaya, Persiaran Bestari, Cyber 11, Cyberjaya 63000, Malaysia; kamaliahmn@gmail.com; 5 Institute for Health Management, Ministry of Health Malaysia, Setia Murni U13/52, Section U13, Setia Alam, Shah Alam 40170, Malaysia; noraziani17@yahoo.com; 6 Institute for Public Health, Ministry of Health Malaysia, Setia Murni U13/52, Section U13, Setia Alam, Shah Alam 40170, Malaysia; drnoorani@moh.gov.my

**Keywords:** missed appointments, children with special needs, developmental disability, caregivers, barriers

## Abstract

Attending appointments is vital for children with special needs, as such appointments involve long-term interdisciplinary care to ensure continuity of care and improve health and well-being. This study was performed to determine the prevalence of missed clinic appointments and identify the factors among those who have ever missed appointments and barriers of access to children’s special needs services at the Child Development Centre (CDC) at the Universiti Kebangsaan Malaysia Medical Centre (UKMMC). Moreover, suggestions for improvement from the caregivers’ perspectives were explored. This is an explanatory sequential mixed methods study among caregivers of children with developmental disabilities aged up to 17 years old. Of 197 caregivers, 62 (31.5%) had missed clinic appointments. Forgetfulness was the most frequently cited reason. The bi-variable analysis showed significant differences in missed appointment rates by gender of caregivers and duration of follow-up. The final logistic regression model demonstrated that, when combined with the effect of being a male caregiver as an independent variable, follow-up duration of more than 6 years increased 2.67 times the risk of missing an appointment. Caregivers’ perceived barriers were transportation, caregiver, child and healthcare services factors. Policies and strategic plans should be focused on key findings from these factors to improve appointment adherence and accessibility to services for children with special needs.

## 1. Introduction

The World Health Organization (WHO) estimates that 15% of the global population has some form of disability, of whom 2 to 4% experience substantial difficulties in functioning [1]. In Malaysia, the National Health and Morbidity Survey 2019 reported that 11.1% of adults and 4.7% of children were found to have disabilities [2]. The need for rehabilitation services has increased swiftly, with contributing factors including the advances in perinatal care (leading to increased survival rates among children with disabilities), variation in the demographic structure of population (increased life expectancy leading to higher proportion of elderly) and lifestyles changes (giving rise to an increase in chronic diseases and disabilities), as well as depression, violence and road-traffic accidents. Earlier detection and improved treatment can increase survival with disabilities, as well as programs that focus on the prevention of disabilities, such as antenatal and postnatal care, child health assessment, school health, immunization, nutrition, injury prevention and healthy lifestyle [3].

In Malaysia, the National Health and Morbidity Survey (NHMS) 2019 reported that 4.7% of children were found to have disabilities [4]. The 2016 NHMS on maternal and child health (MCH) was a landmark survey in which several topics relating to maternal, as well as child health and development, were explored for the very first time on a nationally representative sample by the Institute for Public Health. Of those children screened for autism with the M-CHAT (Modified Checklist for Autism in Toddlers), 1.6% failed the screening test, a higher prevalence as compared to 0.6%, as reported in a feasibility study in 2005 [5]. In addition, the 2016 NHMS reported a slightly higher prevalence among male toddlers, that 20% had not been screened with the M-CHAT prior to this and that a total of 22 children who failed M-CHAT previously were not referred for further assessment and intervention. From the NHMS 2016, the prevalence of developmental delay in children aged 6–59 months was 3.3%, while globally, the estimated prevalence for developmental delay among infants, toddlers and preschool children was 5 to 16% [6]. Screening and referral by front liners in primary healthcare settings are indeed crucial elements. Determining issues associated with barriers and challenges to access to children’s special needs services should be the basis of public health interventions in order to reduce missed appointments and delay in developmental screening and further assessments among children with special needs. However, there is an information gap in regard to the underlying factors for the lower screening or missed appointments among children with special needs, especially in our local setting. All of these issues need to be addressed to avoid negative consequences of missed appointments or non-attendance among these children with special needs.

Missed appointments are a main source of preventable inefficiency that has impacts on the patient health and treatment outcomes [7]. Attending outpatient clinic for treatment is important for children with special needs, since many require long-term interdisciplinary care. Current advances in early intervention have shown that, if intervention is not carried out during critical periods of early neurological and social development, this has prognostic implications [8]. Consistency in intensity and maintenance of health intervention services has been linked to outcome [9]. In addition to affecting patient outcome, missed appointments or appointment absenteeism generally has negative impact on the outpatient clinic, healthcare system and public health. Missed appointments are associated with poor utilization of personnel, reduced economic stability and an overall decline in the quality of clinical services in the outpatient clinic setting. For the healthcare system, patient absenteeism leads to increased healthcare utilization and insurance premiums, and lost profits, encroaching upon public health by draining valuable healthcare resources. While much has been written about inpatient and outpatient attendance in general, there is less information on outpatient appointment attendance in pediatric developmental-disabilities clinics [10]. Hence, this study is important to explore factors associated with missed appointments and barriers and challenges faced by the parents and caregivers to access children’s special needs services in healthcare settings in Malaysia, as well as exploring the parents’ viewpoints on how they could have been helped to improve this.

The objective of this study is to determine the prevalence of missed clinic appointment and to identify its factors, and among those who ever missed appointments, we explore the barriers of access to children’s special needs services at the Child Development Centre in UKMMC and suggestions for improvement from the caregivers’ perspectives. The information gathered is essential to determine the issues associated with barriers and challenges to access the children’s special needs services in healthcare settings so that better intervention strategies and programs can be developed. This can be viewed as a window of opportunity to prevent delay in screening in the future and to lower the rates of developmental-delay screening in this children’s special needs group. Given the above, there is a need to address these issues in the local context.

## 2. Materials and Methods

This is an explanatory sequential mixed methods study among caregivers of children with special needs who had developmental disabilities and were aged up to 17 years old, attending the Child Development Centre (CDC) at the Universiti Kebangsaan Malaysia Medical Centre (UKMMC) from February until May 2021. This CDC was initiated in 2002, and the centre was fully operational in the year 2006. It was the first CDC of its kind in Malaysia that caters to the assessment, diagnosis and intervention of children with special needs. A majority of the children seen in this centre were diagnosed with developmental disabilities, including global developmental delay (GDD), Down’s syndrome (DS), attention deficit hyperactivity disorder (ADHD), autism spectrum disorder (ASD), intellectual disability (ID) and specific learning disability (LD). In 2020, there were about 1011 follow-up cases with developmental disabilities with the above diagnoses seen at the CDC in UKMMC [11].

The primary outcome for this study was the prevalence, expressed as a percentage, of missed clinic appointments at the Child Development Centre at the UKMMC. According to a report by the Diagnostic CDC Census UKMMC 2020, on average, about 84 children with developmental disabilities up to 17 years old were seen monthly in the Child Development Centre in 2020. A study by Kalb et al. (2012) [10] on the determinants of appointment absenteeism at an outpatient Pediatric Autism Clinic reported the absenteeism rate of about 15%. Given the prevalence of 15% and precision of 0.05 at 95% confidence level, the sample size required was calculated to be 196 respondents [12].

### 2.1. Selection and Eligibility

The eligibility criteria were Malaysian caregivers of children aged up to 17 years old who had been diagnosed with a developmental disability by certified medical professionals and attending the Child Development Centre at the the UKMMC, and these caregivers were the biological mother/father, stepmother/father, adopted mother/father or guardian who cared for the children with learning disability. The term “caregiver” describes a person from the above category who showed up with the child during follow-up. Those who were not able to read and understand *Bahasa Melayu* or English, were not able to give consent or were unable to answer the questionnaire due to disabilities were excluded from this study. The principal investigator recruited eligible caregivers by using a convenient sampling method from a registry list of those who came for follow-up appointments at the Child Development Centre at the UKMMC. Study participation was voluntary among those who had given informed consent and did not impact the care their children received, and anonymity was preserved. From 215 eligible caregivers recruited, 197 agreed to participate in this study. There were two phases: initial quantitative phase, using a cross-sectional study design, followed by a qualitative phase, using an explanatory approach. The qualitative study used a purposive sampling method based on the nature of the explanatory sequential mixed methods design [13]. Caregivers of children with developmental disabilities followed up at the CDC who missed an appointment after participating in Phase I of the quantitative study were selected to take part in Phase II of the qualitative study. This method of sampling aids in generating a homogenous sample of participants who have missed an appointment at the Child Development Centre of the UKMMC [14]. The eligibility criteria for the qualitative study (Phase II) were caregivers who participated in the quantitative study (Phase I) who missed an appointment at the Child Development Centre of the UKMMC. Caregivers who did not consent to participate in the qualitative study (Phase II) were excluded. In this study, a sample of 23 caregivers was selected in the qualitative study (Phase II) after data saturation was reached.

### 2.2. Data Collection

During the quantitative study (Phase I), a face-to-face interview, using a questionnaire, was conducted. The questionnaire consisted of two parts: Part A—Sociodemographic Background, and Part B—Child with Developmental Disabilities Information. For Part A, the questions were formulated to provide the sociodemographics and general information of the respondents. The variables of interest for analytical statistics from this section were the district place of stay, distance from centre, age, gender, ethnicity, marital status, level of education, occupation, household income, number of children, medical history and health-insurance coverage. Distance from centre refers to distance from place of stay to the Child Development Centre, UKMMC in kilometer (km). Total household income was reported in RM (Ringgit Malaysia) and classified into 3 categories, B40 (RM 4850 and below), M40 (RM 4851–10,970) and T20 (RM 10,971 and above) [15]. Part B included questions to assess information about the child. The variables of interest for analytical statistics from this section were age of child, gender, ethnicity, status, birth order, diagnosis, presence of house maid or home helper, type of gross movement medical conditions, duration since first appointment at the Child Development Centre, whether the same healthcare providers were seen at every appointment, any missed appointments previously, and the reasons for missed appointments. As for the qualitative study (Phase II), a semi-structured interview protocol was adapted from the literature and developed after the results of Phase I quantitative study was analyzed [16]. The interview protocol was developed jointly by F.F., I.B.I., N.K.N., J.I., A.M.T., K.M.N. and N.K. The interview protocol includes questions regarding the barriers to access children’s special needs services, further details on the caregivers’ barriers and suggestions to improve access to services for children with special needs.

### 2.3. Data Analysis

#### 2.3.1. Quantitative Study (Phase I)

Data were analyzed by using IBM “Statistical Package for Social Sciences” (SPSS) Statistics software version 26.0. The dependent variable, missed appointment, was defined as at least one missed appointment at the Child Development Centre in UKMMC. The bi-variable analysis (Chi-Square test and Fisher’s Exact test) were used to test for significant association between variables. Significant level was considered at *p*-values less than 0.05. Multivariable logistic regression analysis was conducted to determine the predictors of missed appointments.

#### 2.3.2. Qualitative Study (Phase II)

This study adopted a reflexive inductive thematic analysis method as a data-analysis strategy, which is generally used in all qualitative designs [17]. Reflexive approaches highlight a flexible coding process, as there is no reference or code book that can be undertaken by researchers [18]. Firstly, reflexive approaches involve collection of similar codes for theme development later. The themes formed should correlate with the central idea which best describes all sub-themes [19]. Inductive analysis refers to methods that mainly use thorough readings of raw data from the verbatim transcript to extract concepts, themes or a model via interpretations made by a researcher [20]. The six phases of reflexive inductive analysis, as described by Braun and Clarke (2006), were conducted until data saturation was achieved. Phase 1 involved reading data several times and familiarization of data [19] by F.F., I.B.I. and N.K.N. In Phase 2, initial codes were generated by F.F., in which codes identify data that were related to the research questions. As the analysis process continued, codes were organized and assembled into potential themes and sub-themes (Phase 3) by F.F. and I.B.I. Themes and sub-themes were reviewed and refined in Phase 4 with regard to the research objectives by F.F., I.B.I. and N.K.N. Phase 5 was the formal definition, involving development of a detailed analysis of each theme and sub-theme F.F., I.B.I., N.K.N., J.I., A.M.T and K.M.N. Phase 6, the final phase to produce a report, involved integration of domain, themes, sub-themes, and verbatim comments, and correlating them to findings from the literature by F.F., I.B.I., N.K.N., J.I., K.M.N., N.K., N.A.A., S.O. and R.I [18,21]. After these six phases, the researchers had to draw inferences from this explanatory sequential mixed methods research. The term “inference” is defined as the integration of findings from both quantitative and qualitative features of mixed methods research [22].

## 3. Results

### 3.1. Quantitative Study (Phase I)

A total of 197 of caregivers were included for analysis during the study period. From these, 62 caregivers (31.5%) had missed clinic appointments. The most frequently cited reason for missing the appointments was forgetfulness in 71.9%, followed by movement restrictions due to the MCO (Movement Control Order) due to the COVID-19 pandemic in 11%, being busy in 10.6%, and sickness in 5.1%. None of the respondents cited financial problems as the reason for missing appointments, while only 1.4% cited transportation problems (Table 1).

The characteristics of the 197 caregivers who met the inclusion criteria are shown in Table 2. Of these 197 caregivers, the ages were between 28 and 54 years, and their mean age was 39.28 (SD = 5.54) years. The majority was in the age group 30–39 years (55.3%), followed by the age group 40–49 years (38.6%), the age group 50–59 years (5.1%) and the age group 20–29 years (1.0%). Among these caregivers, 78.7% (*n* = 155) were females and 21.3% were males (*n* = 42). For ethnicity, 154 (78.2%) were Malays; 32 (16.2%) were Chinese, eight (4.1%) were Indians; and three (1.5%) reported their ethnicity as “Others”, which were Sino-Native, Dusun and Rungus from Sabah. The majority of them, 156 (79.2%), had completed tertiary education, followed by 40 (20.3%) who had secondary school education and only one (0.5%) with primary school education. A majority of the caregivers are employed in the formal sector, 35.0% in the private sector and 32.5% in the government or semi-government sectors. About 24.9% were unpaid workers or homemakers and only 7.6% were self-employed. In this study, 57 (28.9%) reported that their total household income was RM 4850 and below, 97 (49.2%) reported income of RM 4851–10,970 and 43 (21.9%) reported income of RM 10,971 and above. With regard to marital status, 192 (97.5%) were “Married”, four (2.0%) were “Divorced” and only one (0.5%) was a “Widow”. Most of these caregivers (*n* = 67, 34.0%) had two children, and 29.9% had three children (mean of 2.65). The majority of the caregivers stayed within 40 km from the Child Development Centre (CDC) in UKMMC, at 88.8%, and only 3.1% stayed 81 km or more away from the CDC. Only four (2.0%) of the caregivers reported that they had a learning disability or mental health problem themselves, and 115 (58.4%) had health insurance coverage.

As for the 197 children, the ages were between 3 and 17 years, and their mean age was 8.2 (SD = 2.69) years (Table 3). The majority was in the age group 7–9 years (50.8%), followed by the age group 0–6 years (25.4%), the age group 10–12 years (16.2%) and the age group 13–17 years (7.6%). Among these children, 81.7% (*n* = 161) were males and 18.3% were females (*n* = 36). For ethnicity, 154 (78.2%) were Malays; 31 (15.7%) Chinese; eight (4.1%) were Indians; and four (2.0%) reported their ethnicity as “Others”, which were also Sino-Native, Dusun and Rungus from Sabah, and Siamese (adopted child; caregiver was Chinese). The majority of them, 98% (*n* = 193), was a biological son or daughter, along with three foster sons or daughters (1.5%) and one adopted son or daughter (0.5%). A majority were eldest (*n* = 59, 29.9%) children, followed by youngest (28.9%), middle (23.9%) and being the only child (17.3%).

With regard to the underlying diagnosis, the majority of the children in this study had autism spectrum disorder (*n* = 144, 73.1%), 20 (10.2%) had attention deficit hyperactivity disorder (ADHD), followed by both global developmental delay (GDD) and specific learning disorder (LD) with 13 (6.6%) each, five (2.5%) with intellectual disability (ID) and two (1.0%) with Down’s syndrome (DS). Amongst children with autism spectrum disorder, 46.7% (*n* = 92) were categorized as mild, with details of case diagnosis shown in Table 4. In this study, 172 (87.3%) can walk on their own without using a walking aid and can climb stairs without holding the rail; 24 (12.2%) can walk on their own without using walking aids, but climb stairs while holding the rail; and one (0.5%) can stand on his/her own and only walks using a walking aid or wheelchair on their own, using hands. Among these 197 children, only 12 (6.1%) had a hired housemaid at home to look after them. When the caregivers were asked how long they had been bringing their children to the Child Development Centre in UKMMC for follow-up, the majority, i.e., 93 (47.2%), answered 36 months or less; 67 (34.0%) for 37–72 months; 27 (13.7%) for 73–108 months; and only 10 (5.1%) for 109–144 months. About 20 (10.2%) of the children saw the same healthcare provider at every appointment.

The bi-variable analysis showed significant differences in missed appointment rates by gender of caregivers and duration of follow-up in Child Development Centre, UKMMC. The missed appointment rates were higher among the male caregivers and those who had been bringing their children to the Child Development Centre, UKMMC, for more than 6 years (Table 5 and Table 6). In the second hierarchical multiple logistic regression model (Step 2), the Wald value [X^2^ (df = 1, *n* = 197) = 10.09, *p* < 0.05] for caregiver gender (1) was significant, with an odds ratio value of 3.246, indicating that being a male caregiver increased the risk of missing appointments by 3.25 times when this variable was combined with a second predictor variable, namely a UKMMC follow-up duration of more than 6 years. Wald values [X^2^ (df = 1, *n* = 197) = 6.34, *p* < 0.05], and an odds ratio value of 2.67 for UKMMC follow-up (1) showed that, when combined with the effect of being a male caregiver independent variable, UKMMC follow-up duration of more than 6 years increased the risk of missing appointments by 2.67 times (Table 7).

### 3.2. Qualitative Study (Phase II)

A sample of 23 caregivers was selected to participate in this phase. Table 8 presents the study sample characteristics in Phase II. The caregivers who participated in Phase II were predominantly aged 30 to 49 years old (95.7%), male (65.2%), Malays (91.3%), had tertiary level of education (95.7%), worked in the private sector (52.2%), were in M40 income group (69.6%), were married (100%), had two children (43.5%), lived within 40 km from the Child Development Centre of UKMMC (95.7%) and who were followed up over a duration of 6 years or less (73.9%).

Caregivers’ narratives gave context-rich data about the barriers that they had to face in order to attend the appointments at the Child Development Centre of the UKMMC for their children. Further questions were asked such as distance, time, cost, facilities and healthcare provider behaviors. Additionally, caregivers suggested several recommendations that they felt could improve their children’s access to services at the Child Development Centre of the UKMMC.

#### 3.2.1. Barriers Accessing Special Needs Services

1. Transportation Factors

Caregivers’ narrative descriptions showed that transportation barriers were often encountered when traveling to the Child Development Centre of the UKMMC. Modes of transportation were individual private transportation, such as cars, and public transportation. For those using personal transportation, caregivers described barriers and challenges associated with parking issues and traffic congestion. The caregivers gave the following descriptions:

*“When my child has appointment here, I have to come early although the appointment time a bit later because I want to avoid traffic jam and difficulty to find parking. If I come late, I cannot find parking here. It is difficult if I have to park far away, and my child and I have to walk so far to the hospital”*.

(Participant 7: 40 years old, Malay, Mother, Child 8 years old with Autism)

*“I have to bring along my wife to the hospital. Then, I dropped them at the hospital lobby first. Later after I parked my car, I joined them for the appointment. I wish hospital has special parking for those who have children with special needs like us”*.

(Participant 12: 36 years old, Malay, Father, Child 12 years old with Global Developmental Delay)

Among those who were using public transportation to attend the Child Development Centre, UKMMC, they reported challenges related to accessibility. One narration shows the caregiver’s opinion:

*“If there is LRT station nearby the hospital, it would be easier for me and my child. I have to take taxi from LRT to the hospital. And not easy for us to get taxi from the LRT”*.

(Participant 5: 42 years old, Malay, Mother, Child 6 years old with Intellectual Disability)

2. Caregiver Factors

Caregivers highlighted their issues and challenges that they faced pertaining to follow-up clinic appointments at the Child Development Centre, UKMMC. It was a conflicting dilemma between their commitment at work and attending appointments, as the long duration needed during appointment attendance was perceived as a barrier to adherence of appointments. For example, caregivers explained their struggle in order to follow the appointment’s slot:

*“I have to take my own annual leave to bring my child here. I really hope if they can increase the frequency of appointment in clinic. If possible, during this pandemic, can do appointment through virtual such as Google Meet”*.

(Participant 2: 45 years old, Malay, Mother, Child 6 years old with Autism)

*“Time consuming, I don’t have any choice but have to take annual leave to bring my child here”*.

(Participant 19: 42 years old, Malay, Mother, Child 7 years old with Autism)

*“It is difficult to apply leave to bring my child for the appointment”*.

(Participant 6: 38 years old, Chinese, Father, Child 5 years old with Global Developmental Delay)

*“You know, I am busy at work. Most of the time, I am on duty with no replacement”*.

(Participant 4: 45 years old, Malay, Mother, Child 8 years old with Global Developmental Delay)

*“Due to work schedule, it is difficult to bring my child on appointment day”*.

(Participant 8: 42 years old, Malay, Mother, Child 9 years old with Autism)

3. Child Factors

This theme represents the competing priorities that arise between their children’s other obligations and the scheduled appointments when these overlap time-wise. Caregivers highlighted that they needed to choose whether to attend their children’s appointment at the Child Development Centre, UKMMC, or to attend other functions of similar importance, such as attending to their other healthcare conditions or appointments related to schooling or school activities. Below, the challenges are highlighted:

*“I have to postpone the appointment last minute because my child was sick. Then, I called the clinic to set new date, but as expected, it took longer time for the next appointment. It’s quite challenging but we have no choice”*.

(Participant 1: 35 years old, Malay, Father, Child 10 years old with Autism)

*“When the appointment schedule fell during the school days, my child has to skip school to attend the appointment. You know, it’s not easy to get the appointment date. So, we have no choice. Luckily, the teachers understand our situation. However, we have to postpone the appointment if our child has examination on the same day”*.

(Participant 23: 42 years old, Malay, Mother, Child 11 years old with Autism)

*“If I were alone, it is difficult for me to handle the child by myself; my child always wandering around the hospital”*.

(Participant 20: 36 years old, Malay, Father, Child 9 years old with Autism)

4. Healthcare Services Factors

Caregivers described the hindrance they met during the appointment scheduling and services at the Child Development Centre, UKMMC. They revealed that inflexibility in setting the appointment slot imposed a constraint. Due to the lack of flexibility, this created conflict in those who are working, whereby they had to take time off in order to attend the appointments.

*“The appointment slots were only in the morning. I am working. If possible, to have appointment slots in the afternoon as well since most of meetings at the office are in the morning. Difficult for me to apply leave to bring my child for appointment. And they should have more slots to reduce long gap before the next appointment”*.

(Participant 6: 38 years old, Chinese, Father, Child 5 years old with Global Developmental Delay)

Caregivers mentioned that there was a lack of reminder notification system, hence leading to problems rescheduling the appointments, especially during the pandemic time, where they had to wait a longer gap time for the next appointment.

*“The hospital should give the appointment reminder earlier at least a week before by WhatsApp or SMS”*.

(Participant 13: 39 years old, Malay, Mother, Child 8 years old with Attention Deficit Hyperactivity Disorder)

Most of the caregivers also informed us that they saw different healthcare providers every time they came. Thus, they had to inform the healthcare provider again about their children’s medical history. They emphasized the need for more healthcare providers and more appointment slots with shorter gaps in between the appointments.

*“Healthcare providers need to read first the case notes before the appointment”*.

(Participant 4: 45 years old, Malay, Mother, Child 8 years old with Global Developmental Delay)

*“We have to wait long time for next appointment. They need to increase the number of healthcare providers”*.

(Participant 14: 35 years old, Malay, Father, Child 8 years old with Autism)

#### 3.2.2. Recommendations to Improve Accessibility to the Special Needs Services

Numerous recommendations regarding accessibility were pointed out that they thought would improve the appointment attendance. Generally, caregivers’ suggestions focused profoundly on transportation factors and healthcare services factor. Under the transportation factor category, caregivers had a few suggestions to improve parking facilities and to have special parking for those who have children with special needs. As for the caregiver and child factors, caregivers proposed that the healthcare providers consider their struggles in keeping with the appointment schedules. Within the healthcare services factor category, caregivers suggested that they be given more flexibility in terms of appointment schedule timing, that the reminder notification system be improved through the provision of numerous modes of communication and that more appointment slots be made available in order to reduce the long gap between follow-up appointments.

## 4. Discussion

The objective of this study was to determine the prevalence of missed appointments and to identify the factors of missed appointments among caregivers attending the Child Development Centre in UKMMC. Among those who ever missed appointments, the barriers of access to children’s special needs services and suggestions for improvement from the caregivers’ perspectives were explored. The results of the final logistic regression model demonstrated that, when combined with the effect of being a male caregiver as an independent variable, UKMMC follow-up duration of more than 6 years increased 2.67 times the risk of missing an appointment.

This CDC is situated in an academic institution, and while it is surrounded by residences for low-, middle- and high-income groups, it also receives referrals from further areas. The centre was chosen because it was the first centre for child development in the capital city, had been running developmental clinics for over 10 years and, thus, is established as a centre of some experience. The centre is in the capital city of Kuala Lumpur and hence in an urban region. With regard to the socioeconomic class served by this centre, a majority (49.2%) of the attendees were from middle-income families, with only 28.9% from families who were more affluent. The patients who attend the clinic were mostly from the Klang Valley, though a small number were from out of state. Almost all, if not all, child development centres in Malaysia are concentrated in cities such as Kuala Lumpur and Penang, which are in urban regions.

In this study, the prevalence of missed appointments at the Child Development Centre at UKMMC was 31.5%, comparable to a study at the pediatric clinic in a Malaysian tertiary hospital [23], but higher than a study at an outpatient Pediatric Autism Clinic in the United States of America [10]. Missed medical appointments have harmful effects on the patient, clinic, healthcare system and, in due course, public health as a whole [24]. Missed appointments are associated with poor utilization of personnel, reduced economic stability and an overall decline in the quality of clinical services in the outpatient clinic setting particularly for children with special needs. Missed appointments could lead to delay in developmental screening and interruption of proper assessment and management for children with special needs. In this study, the most frequently cited reason among the caregivers for missing the appointments was forgetfulness, followed by movement restriction due to the MCO (Movement Control Order) resulting from the COVID-19 pandemic, being busy and sickness, while only a few mentioned transportation problems.

This study was performed during the COVID-19 pandemic and the Malaysian Government–imposed Movement Control Order (MCO), which affected many aspects of hospital outpatients’ care, including limitations and restrictions of the number of patients seen during clinic hours and travel restrictions across regions. In addition, the pediatric department of the study centre was in the process of moving to a new children’s hospital, and this also affected services. The caregivers indicated to clinic staff when reminded of clinic appointments that they were afraid of COVID-19 exposure to their children and themselves while bringing them to the hospital. The COVID-19 pandemic significantly reduced the number of routine outpatient visits since many patients were also avoiding visits as they do not want to leave their homes and risk exposure to the disease [25]. This finding was congruent with the National pulse survey on continuity of essential health services during the COVID-19 pandemic whose objectives were to swiftly assess the extent of the impact of the COVID-19 pandemic on health systems and essential health services during the course of the pandemic [26]. In this survey, 94% of the 135 participating countries and territories reported some kind of service disruption during the preceding three months (January–March 2021), only slightly reduced from the percentage of countries reporting service disruptions in the first pulse survey rounds during quarters three and four of 2020. Primary care, rehabilitative, palliative and long-term care were most deeply affected, with more than 40% of countries reporting disruptions affecting the availability of and access to quality services, including for the most susceptible individuals. In some countries, measures for COVID-19 control may contribute to increased barriers to accessing care (for example: limitations in movement, fear of getting infected, limited personal protective equipment or access, loss of income and increased financial burden). However, despite this challenging pandemic scenario, the absenteeism rate observed in this study was still comparable to rates during non-pandemic times, as shown in a study performed by Jamil et al. 2011 [23]. This suggests the caregivers were trying to keep to the children’s appointments even in difficult circumstances, and the clinic was also attempting to maintain the schedule of follow-ups for children as rigorously as possible.

In this study, the main reason for missed appointments among the caregivers was forgetfulness. This is consistent with studies by Jamil et al. (2011) [23] and Cronin et al. (2018) [27] and highlights the importance of reminders prior the clinic appointment to enable better turnout, as was described in several other studies [28,29]. It also emphasizes the need for parents to find more effective ways to keep to appointments, as these follow-up appointment dates are given a number of months earlier. The second most common reason for missed appointments was due to the MCO (Movement Control Order). During the COVID-19 pandemic, most of the appointments were rescheduled either from the hospital side or by the caregivers themselves. The MCO announcements were being made near the appointment dates, in which caregivers needed to arrange and settle other matters related to the MCO; hence, they would sometimes miss their appointments. Due to the restrictions from the MCO, resulting in difficulty to cross the borders, those who were living outside of the hospital’s district and state especially had difficulties attending their appointments. Another reason for missing the appointment was the fact that caregivers were too busy. A possible explanation for this is that the caregivers placed a higher priority on other activities compared to bringing their children to attend clinic appointments. It is important for the caregivers to prioritize their children’s appointment visits, and, to this end, health practitioners may need to provide explanation so that caregivers can understand why their children should attend follow-ups, especially those with special needs. Caregivers may lack a sense of urgency and responsibility, and they may not regard missing appointments as vital to their children’s care and to healthcare providers [30]. A study by Ofei-Dodoo et al. (2019) emphasized potential ways to reduce missed appointments by educating patients on how to cancel an appointment if they already know that they cannot make it on the day of appointment [31]. Basic means of canceling appointments should be practiced, for example, sending a text message or calling the clinic to cancel. Information about missed appointments might be valuable in the initial identification of patients who are at risk for medical negligence. Parental participation is a crucial element of their children’s care. Even though children may take part more in their treatment as they mature, parents still play a significant role especially during childhood and adolescence [32].

Our analysis showed that being a male caregiver was the most significant predictor of missed appointments in this study. There was a paucity of research studies assessing the impact of caregiver’s sex on children’s absenteeism in clinic. Findings by Corfield et al. (2008) revealed that male patients were significantly more likely to fail to attend clinic appointment, as opposed to women [33]. In another study, similar findings were reported, where significantly more men than women failed to keep their appointments [34]. One hypothesis is that male caregivers may perceive that they have greater work obligations as a primary breadwinner and therefore may spend less time with the children compared to mothers, and they might also perceive the children’s condition as mild or not concerning.

Another significant predictor was outpatient clinic follow-up duration of more than 6 years. This finding was also consistent with a study by Kalb et al. (2012) at an outpatient Pediatric Autism Clinic in USA which demonstrated that increased duration of follow-up appointments was the predictor for missed appointments among the respondents [10]. It is possible that, after a certain amount of time being followed-up, medical concerns have lessened considerably, therefore reducing priority and urgency to attend the appointments, and also the children possible getting busier with other activities. This shows that the longer duration of appointment follow-ups increased the likelihood of missing appointments [35].

This study also explored caregivers’ perceived barriers in accessing the special needs services for the children. In this study, qualitative design assessed the challenges faced by the caregivers in relation to appointment adherence. The open-ended method permitted caregivers to recognize the related fundamentals that could be improved to smoothen the appointment adherence and, hence, in some way, reduce the prevalence of missed appointments. Though the delivery of children with special needs services has advanced progressively over the years with regard to types of treatment offered and effective methods, it is vital to understand the impact of missing appointments and its consequences for children’s general welfare. Caregivers in this study highlighted barriers related with transportation. Earlier studies have also demonstrated transportation barriers to appointment adherence, including parking issues [21,36,37]. Besides transportation, caregiver and child factors were identified as barriers to appointment adherence in this study. Comparable to our findings, arrangements that conflicted with employment commitments and other school-related activities were also reported [21,31,38]. Barriers to appointment adherence which were associated with healthcare services were demonstrated in other studies, including lack of flexibility in appointment scheduling [39], inadequate appointment reminders [40] and a long gap in between the appointment schedule and the exact appointment date [21,41].

Exploring caregivers’ recommendations to improve the healthcare system based on their experience is an established way to develop effective holistic family-centred interventions and strategies to sustain appointment adherence [21]. A few suggestions were given to improve parking facilities and special parking for those who have children with special needs. Transportation support was identified as a way to facilitate appointment adherence such as ease of parking, having a car, improved transport links and accessibility to public transit [21,42,43]. Caregivers also proposed healthcare providers be more flexible in assisting them to keep their appointment schedules, by giving some leeway with regard to scheduling. Ballantyne et al. 2019 [21] described other studies which emphasized how parents themselves could address their competing priorities and how parental motivation could improve their children’s health appointment attendance [42,43]. As mentioned earlier, educating parents about the importance of attending appointments and effects of missing appointments could influence appointment adherence [42]. Parents are actually in control of most aspects of their situation and thus responsible for appointment adherence, which can be empowered to do. Supporting the family in their healthcare and treatment encourages a constructive family-centred setting that will, in due course, lead to better outcomes [21].

Caregivers recommended for more flexibility in appointment schedule timing, whereby some noted that they would prefer for clinics to be in the afternoon. However, this may not be optimal for observation of children at their best in terms of development, as healthcare providers would want to see the children when they are still fresh in the earlier part of the day and seeing them in the afternoon may impact their performance, especially if they have had a busy day. Caregivers in this study also proposed alternative sites and service delivery models of care such as virtual approaches that permits providing health services to those who cannot attend physically. One of the recommendations from the caregiver was to have the appointments through virtual platforms such as Google Meet, if possible, during the pandemic. However, it is difficult to perform virtual assessments for certain developmental domains such as social interactions, especially for children with special needs. The nurses do send reminders a week before appointment via the phone text messaging system. In fact, in this centre, the CDC is the only pediatric clinic that sends reminders for appointments. It is possible that caregivers did not check their message, or there may have been delay in the delivery. In the future, suggestions to improve the reminder notification system through numerous modes of communications will be looked into.

During the COVID-19 pandemic, the hospital needed to cut down the numbers of patients attending clinic, so as to not endanger the family and staff and reduce COVID-19 exposure, in keeping with the Standard of Procedure (SOP) implementation of physical distancing. In addition, during the data collection taking place, they were in the midst of transferring to a new children’s hospital. This is partly why appointments were more spaced apart. In certain cases, seeing the patients earlier might not give enough time to see meaningful changes in development. Moreover, when they missed the appointment, the clinic has to find new slot for them which is outside of normal scheduling for all patients. The clinic’s schedule opens during regular office hours, and as this is not a private sector, it does not have resources to open during weekends and evenings.

Most caregivers preferred to see the same healthcare providers during each follow-up in order to avoid repeating their children’s medical history. They requested for more healthcare providers and more appointment slots with shorter gaps in between the appointments’ dates. Different healthcare providers are not unexpected in this setting because this is a teaching hospital and there is a rotation of training doctors who are being trained in this area. The attending doctors also have other commitments and need to attend inpatients on the wards as well. As a teaching hospital, it is the responsibility of the teaching staff to teach pediatric trainees in pediatrics and in the developmental pediatrics specialty. As a training centre, this is part of the process of training, and thus increasing the number of doctors in this specialty is of utmost importance, and indirectly addresses the caregivers’ requests for more practitioners. Developmental pediatrics is very much in its infancy in Malaysia. There are currently less than 20 fully recognized developmental pediatrics. Hence, we need more trained pediatricians in developmental pediatrics. It is hoped in the future that there will be more Developmental Pediatrics doctors to cater for these children with special needs.

The strength and uniqueness of this study is that it is one of the first mixed methods study that was conducted locally and internationally to determine the prevalence and factors of missed appointments among the caregivers of children with special needs who had developmental disabilities and to explore barriers of access to children’s special needs services and suggestions for improvement from the caregivers’ perspectives. To the best of our knowledge, there are very few studies in this area especially in children with special needs. During the study, the same interviewer, the principal investigator, was involved in the interviewing session to ensure accuracy and uniformity of the information attained. In this study, explanatory sequential mixed methods study design aided to deeply explore the factors of missed appointments among caregivers and barriers with regard to access children’s special needs services. To our understanding, this is one of the first research studies to assess missed appointment and related barriers of appointment adherence within child development centre settings among caregivers of children with multiple developmental disabilities diagnoses. Even though some of the barriers and challenges found in this study were also documented in previous studies, there are a limited number of works in the published literature that are related to the experience of caregivers of children with multiple developmental disabilities similar to our setting.

With regard to limitations, reporting bias may occur as reliability of results from the quantitative part of this study relies on the truthfulness during answering. Response bias may occur as they might give answers according to what the researcher hoped for. Other limitations include potential selection bias, which may limit the generalizability of the study to only those managed in urban-based hospital, which is where the study was conducted. Our study sample was recruited from one setting and, hence, may not be representative of the general population of caregivers of children with special needs with developmental disabilities. Recall bias is also another consideration especially when the caregivers were asked to recall how long they had been bringing their children to the CDC for follow-up. Some caregivers may have difficulty in recalling this type of information and it may lead to under or over-reporting, resulting in information bias. Overall, it is hoped that the qualitative part of this study is able to compensate for limitations in the quantitative component [18,44].

## 5. Conclusions

In summary, this study signifies an imperative step toward understanding missed appointments and factors associated with it, barriers of access to child development centres among children with developmental disabilities and suggestions for improvement from the caregivers’ perspectives. The results indicated that the missed-appointments rate is high and the main reason was forgetfulness. The predictors for missed appointments were being male caregivers and those who had been bringing their children for follow-up for more than 6 years. This study utilized an in-depth understanding of caregivers’ perspectives in order to develop a better grasp of the factors affecting appointment adherence. We hope that the findings from this study contribute to the research literature on accessibility of healthcare provisions in children with developmental disabilities. Strategies should be focused on key findings from the identified factors of transportation, caregiver, child and healthcare services to improve appointment adherence. The evidence gained from this research can be utilized to develop policies and strategic plans to ensure that these children receive the full range of healthcare services they are entitled to.

## Figures and Tables

**Table 1:** Causes of missed appointment as mentioned by the caregivers.

Variables	Frequency, *n*	Percentage (%)
Forget	156	71.9
Sickness	11	5.1
Busy	23	10.6
Transportation Problem	3	1.4
Financial Problem	0	0.0
MCO	24	11.0
Total Response, N	217	100.0

**Table 2:** Sociodemographic and characteristics of caregivers (*n* = 197).

Variables		*n*	Percentage (%)	Mean	SD
All		197	(100.0)		
Age (years)				39.28	5.54
	20–29	2	(1.0)		
	30–39	109	(55.3)		
	40–49	76	(38.6)		
	50–59	10	(5.1)		
Gender					
	Male	42	(21.3)		
	Female	155	(78.7)		
Ethnicity					
	Malay	154	(78.2)		
	Chinese	32	(16.2)		
	Indian	8	(4.1)		
	Others	3	(1.5)		
Education Level					
	Up to Standard 6	1	(0.5)		
	Form 1–5	40	(20.3)		
	Form 6 or College/Diploma	62	(31.5)		
	University Degree and above	94	(47.7)		
Employment					
	Government/Semi	64	(32.5)		
	Private	69	(35.0)		
	Self-Employed	15	(7.6)		
	Unpaid Workers or Homemaker	49	(24.9)		
Household Income (monthly)				7790.36	5200.26
	B40 (≤RM 4850)	57	(28.9)		
	M40 (RM 4851–10,970)	97	(49.2)		
	T20 (≥RM 10,971)	43	(21.9)		
Marital Status					
	Married	192	(97.5)		
	Divorced	4	(2.0)		
	Widow	1	(0.5)		
Number of Children				2.65	1.16
	1	30	(15.2)		
	2	67	(34.0)		
	3	59	(29.9)		
	4	28	(14.2)		
	5	9	(4.6)		
	6	4	(2.0)		
Distance (km)				24.93	42.71
	≤40	175	(88.8)		
	41–80	16	(8.1)		
	≥81	6	(3.1)		
Learning Disability/Mental Health Problem					
	Yes	4	(2.0)		
	No	193	(98.0)		
Health Insurance					
	Yes	115	(58.4)		
	No	82	(41.6)		

**Table 3:** Sociodemographic and characteristics of children with developmental disabilities (*n* = 197).

Variables		*n*	Percentage (%)	Mean	SD
All		197	(100.0)		
Age (years)				8.20	2.69
	0–6	50	(25.4)		
	7–9	100	(50.8)		
	10–12	32	(16.2)		
	13–17	15	(7.6)		
Gender					
	Male	161	(81.7)		
	Female	36	(18.3)		
Ethnicity					
	Malay	154	(78.2)		
	Chinese	31	(15.7)		
	Indian	8	(4.1)		
	Others	4	(2.0)		
Child Status					
	Biological Son/Daughter	193	(98.0)		
	Stepson/Daughter	0	(0.0)		
	Adopted Son/Daughter	1	(0.5)		
	Foster Son/Daughter	3	(1.5)		
Birth Order					
	Only Child	34	(17.3)		
	Eldest	59	(29.9)		
	Middle	47	(23.9)		
	Youngest	57	(28.9)		
Diagnosis					
	Global Development Delay	13	(6.6)		
	Down’s Syndrome	2	(1.0)		
	Attention Deficit Hyperactivity Disorder	20	(10.2)		
	Autism	144	(73.1)		
	Intellectual Disability	5	(2.5)		
	Specific Learning Disorder	13	(6.6)		
Hiring Maid					
	Yes	12	(6.1)		
	No	185	(93.9)		
Gross Movement					
	Can walk and climb stairs on their own	172	(87.3)		
	Can walk on their own but climb stairs holding rail	24	(12.2)		
	Can sit on their own but does not walk without support	1	(0.5)		
UKMMC Follow-Up Duration (month)				48.42	31.45
	≤36	93	(47.2)		
	37–72	67	(34.0)		
	73–108	27	(13.7)		
	109–144	10	(5.1)		
Same Healthcare Provider					
	Yes	20	(10.2)		
	No	177	(89.8)		
Missed UKMMC Appointment					
	Yes	62	(31.5)		
	No	135	(68.5)		

**Table 4:** Diagnosis characteristics of children with developmental disabilities (*n* = 197).

	Severity	Mild		Moderate		Severe		Total	
Diagnosis		*n*	(%)	*n*	(%)	*n*	(%)	*n*	(%)
Global Developmental Delay (GDD)		8	(4.1)	5	(2.5)	0	(0.0)	13	(6.6)
Down’s Syndrome (SD)		1	(0.5)	1	(0.5)	0	(0.0)	2	(1.0)
Attention Deficit Hyperactivity Disorder (ADHD)		15	(7.6)	5	(2.5)	0	(0.0)	20	(10.2)
Autism		92	(46.7)	38	(19.3)	14	(7.1)	144	(73.1)
Intellectual Disability (ID)		1	(0.5)	2	(1.0)	2	(1.0)	5	(2.5)
Specific Learning Disorder (SLD)		Dyscalculia		Dysgraphia		Dyslexia			
		*n*	(%)	*n*	(%)	*n*	(%)		
		0	(0.0)	2	(1.0)	11	(5.6)	13	(6.6)
Total All, N								197	(100)

**Table 5:** Differences in missed appointment rates by sociodemographic and characteristics of caregivers in the study (*n* = 197).

			Appointment				
Variables		Total, *n*	Ever Missed *n* (%)		Never Missed *n* (%)		*p*-Value *
All		197	62	(31.5)	135	(68.5)	-
Age (years)							0.550
	<40	111	33	(29.7)	78	(70.3)	
	≥40	86	29	(33.7)	57	(66.3)	
Gender							0.004 *
	Male	42	21	(50.0)	21	(50.0)	
	Female	155	41	(26.5)	114	(73.5)	
Ethnicity							0.862
	Malay	154	48	(31.2)	106	(68.8)	
	Non-Malay	43	14	(32.6)	29	(67.4)	
Education Level							0.472
	Primary/Secondary	41	11	(26.8)	30	(73.2)	
	Tertiary	156	51	(32.7)	105	(67.3)	
Employment							0.543
	Government/Semi/Private	133	40	(30.1)	93	(69.9)	
	Self/Unpaid Workers/Homemaker	64	22	(34.4)	42	(65.6)	
Household Income (monthly)							0.569
	B40 (≤RM 4850)	57	21	(36.8)	36	(63.2)	
	M40 (RM 4851–10,970)	97	29	(29.9)	68	(70.1)	
	T20 (≥RM 10,971)	43	12	(27.9)	31	(72.1)	
Marital Status							0.651
	Married	192	60	(31.2)	132	(68.8)	
	Non-Married	5	2	(40.0)	3	(60.0)	
Number of Children							0.696
	1	30	9	(30.0)	21	(70.0)	
	2	67	19	(28.4)	48	(71.6)	
	3	59	18	(30.5)	41	(69.5)	
	≥4	41	16	(39.0)	25	(60.1)	
Distance (km)							0.653
	≤40	175	56	(32.0)	119	(68.0)	
	>40	22	6	(27.3)	16	(72.7)	
Learning Disability/Mental Health Problem							1.000
	Yes	4	1	(25.0)	3	(75.0)	
	No	193	61	(31.6)	132	(68.4)	
Health Insurance							0.495
	Yes	115	34	(29.6)	81	(70.4)	
	No	82	28	(34.1)	54	(65.9)	

* Significant at *p* < 0.05.

**Table 6:** Differences in missed appointment rates by sociodemographic and characteristics of children with developmental disabilities in the study (*n* = 197).

			Appointment				
Variables		Total, *n*	Ever Missed *n* (%)		Never Missed *n* (%)		*p*-Value *
All		197	62	(31.5)	135	(68.5)	-
Age (years)							0.061
	3–9	150	42	(28.0)	108	(72.0)	
	10–17	47	20	(42.6)	27	(57.4)	
Gender							0.086
	Male	161	55	(34.2)	106	(65.8)	
	Female	36	7	(19.4)	29	(80.6)	
Ethnicity							0.862
	Malay	154	48	(31.2)	106	(68.8)	
	Non-Malay	43	14	(32.6)	29	(67.4)	
Child Relationship							0.592
	Biological Son/Daughter	193	60	(31.1)	133	(68.9)	
	Non-Biological Son/Daughter	4	2	(50.0)	2	(50.0)	
Birth Order							0.776
	Only Child	34	10	(2.4)	24	(70.6)	
	Have Siblings	163	52	(31.9)	111	(68.1)	
Diagnosis							0.814
	Autism	144	46	(31.9)	98	(68.1)	
	Other Diagnosis	53	16	(30.2)	37	(69.8)	
Hiring Maid							0.523
	Yes	12	5	(41.7)	7	(58.3)	
	No	185	57	(30.8)	128	(69.2)	
Movement							0.602
	Without Assistance	172	53	(30.8)	119	(69.2)	
	With Assistance	25	9	(36.0)	16	(64.0)	
UKMMC Follow-Up Duration							0.035 *
	≤6 years	160	45	(28.1)	115	(71.9)	
	>6 years	37	17	(45.9)	20	(54.1)	
Same Healthcare Provider							0.386
	Yes	20	8	(40.0)	12	(60.0)	
	No	177	54	(30.5)	123	(69.5)	

* Significant at *p* < 0.05.

**Table 7:** Multiple logistic regression model to predict missed appointment among the caregivers in the study population (*n* = 197).

Variables	B	SE	Wald	df	Sig.	Odds Ratio	95% CI for Odds Ratio	
							Lower	Upper
Step 1								
Caregiver Gender (1)	1.023	0.358	8.145	1	0.004	2.780	1.378	5.612
Constant	0.000	0.309	0.000	1	1.000	1.000		
Step 2								
Caregiver Gender (1)	1.178	0.371	10.087	1	0.001	3.246	1.570	6.715
UKMMC Follow-Up (1)	0.981	0.390	6.337	1	0.012	2.666	1.242	5.720
Constant	−0.892	0.475	3.532	1	0.060	0.410		

**Table 8:** Sociodemographic and characteristics of caregivers in Qualitative Study Phase II (*n* = 23).

		*n*	Percentage (%)
All		23	(100.0)
Age (years)			
	30–39	12	(52.2)
	40–49	10	(43.5)
	50–59	1	(4.3)
Gender			
	Male	15	(65.2)
	Female	8	(34.8)
Ethnicity			
	Malay	21	(91.3)
	Chinese	2	(8.7)
Education Level			
	Form 1–5	1	(4.3)
	Form 6 or College/Diploma	12	(52.2)
	University Degree and above	10	(43.5)
Employment			
	Government/Semi	8	(34.8)
	Private	12	(52.2)
	Self–Employed	2	(8.7)
	Unpaid Workers/Homemaker	1	(4.3)
Household Income (monthly)			
	B40 (≤ RM 4850)	2	(8.7)
	M40 (RM 4851–10970)	16	(69.6)
	T20 (≥ RM 10971)	5	(21.7)
Marital Status			
	Married	23	(100.0)
Number of Children			
	1	1	(4.3)
	2	10	(43.5)
	3	6	(26.1)
	4	6	(26.1)
Distance (km)			
	≤40	22	(95.7)
	41–80	1	(4.3)
UKMMC Follow-Up Duration (month)			
	≤36	6	(26.1)
	37–72	11	(47.8)
	73–108	4	(17.4)
	109–144	2	(8.7)

## Data Availability

Due to the nature of this research, privacy and ethical concerns, participants of this study did not agree for their data to be shared publicly, so neither the data nor the source of the data can be made available.
